# Does early-life respiratory syncytial virus infection induce epigenetic changes that promote asthma development?

**DOI:** 10.3389/falgy.2026.1837534

**Published:** 2026-05-26

**Authors:** Sara Pischedda, Alberto Gómez-Carballa, Federico Martinón-Torres, Antonio Salas

**Affiliations:** 1 Unidade de Xenética, Instituto de Ciencias Forenses, Facultade de Medicina, Universidade de Santiago de Compostela, Santiago de Compostela, Galicia, Spain; 2 Genética de Poblaciones en Biomedicina (GenPoB) Research Group, Instituto de Investigación Sanitaria (IDIS), Hospital Clínico Universitario de Santiago (SERGAS), Santiago de Compostela, Galicia, Spain; 3 Genetics, Vaccines and Infections Research Group (GenViP), Instituto de Investigación Sanitaria de Santiago, Universidade de Santiago de Compostela, Santiago de Compostela, Galicia, Spain; 4 Centro de Investigación Biomédica en Red de Enfermedades Respiratorias (CIBER-ES), Madrid, Spain; 5 Translational Pediatrics and Infectious Diseases, Department of Pediatrics, Hospital Clínico Universitario de Santiago de Compostela, Santiago de Compostela, Galicia, Spain

**Keywords:** asthma, epigenomics, gene-expression, methylome, respiratory syncytial virus, respiratory viral infection, transcriptome

## Abstract

Respiratory viral infections in early life are strongly associated with the development of childhood asthma, although the mechanisms linking infection to long-term respiratory outcomes remain unclear. It is not yet established whether viral infections directly cause persistent airway damage or instead unmask an underlying host susceptibility. Increasing evidence suggests that epigenetic mechanisms, particularly DNA methylation, may act as a biological interface connecting environmental exposures with immune and respiratory development. Epigenome-wide association studies have identified consistent DNA methylation signatures associated with childhood asthma, suggesting that altered immune programming may precede clinical disease rather than simply reflecting established inflammation. Our recent work in children hospitalized with respiratory syncytial virus (RSV) infection provides additional insights into the role of the epigenome in shaping later respiratory sequelae. In a longitudinal study, DNA methylation profiles obtained during the acute phase of infection were associated with subsequent wheezing and asthma development. In this Perspective, we explore the hypothesis that early-life RSV infection may contribute to epigenetic changes that increase the risk of developing asthma later in life. By examining DNA methylation biomarkers previously associated with asthma in our cohort of RSV-infected children, we observed that individuals who later developed asthma showed more alterations in these biomarkers, predominantly hypomethylation patterns consistent with prior asthma studies. These proof-of-concept findings suggest that RSV infection may initiate asthma-related epigenetic mechanisms. Further longitudinal studies are needed to determine whether these changes reflect pre-existing susceptibility or infection-induced epigenetic remodeling, and to clarify their persistence and functional impact on immune and airway biology.

## Introduction

Respiratory syncytial virus (RSV) is one of the most common respiratory viruses affecting infants and young children worldwide and represents one of the leading causes of acute lower respiratory infections (LRTI) during early life (1). The global burden of RSV is considerable, with millions of infections occurring annually and particularly high morbidity and mortality in children younger than five years (2). In addition to its high incidence, RSV infection is associated with a wide spectrum of clinical severity, ranging from mild respiratory symptoms to severe bronchiolitis requiring hospitalization, with genetic host-related factors contributing significantly to disease variability (3).

Beyond the acute phase of infection, increasing evidence suggests that RSV infection during infancy may be associated with long-term respiratory sequelae. Several epidemiological studies have reported an association between early RSV infection and the subsequent development of recurrent wheezing and childhood asthma (4–6). Children who experience RSV-associated LRTI have been estimated to have a two- to twelve-fold increased risk of developing asthma later in childhood compared with children without severe RSV disease (7). Although this association has been consistently explored across observational studies and systematic reviews, the precise nature of this relationship remains debated (7–9), particularly regarding causality and underlying biological mechanisms.

One of the key unresolved questions is whether viral infections directly contribute to the pathogenesis of asthma by inducing persistent alterations in the respiratory tract or whether severe infections occur preferentially in children who are already predisposed to asthma due to underlying genetic or immunological susceptibility. Distinguishing between these two possibilities is critical to understanding disease mechanisms and developing preventive strategies.

According to the World Health Organization (WHO), asthma represents one of the most common chronic respiratory diseases in children, affecting approximately 14% of the pediatric population (10). The prevalence of childhood asthma has increased over the past decades, and this could be explained by genetic factors and environmental exposures during early life. Among the potential modifiable environmental factors, viral respiratory infections have been consistently identified as major contributors to wheezing illnesses in early childhood and as potential determinants of asthma development (11). Recurrent wheezing in infancy is common, and in nearly half of cases, it is a strong predictor of asthma development later in life (12). Asthma pathogenesis involves immune-mediated inflammation and complex interactions between genetic, epigenetic, and environmental factors that ultimately lead to airway remodeling and airway hyperresponsiveness. In recent years, epigenetic mechanisms have emerged as potential mediators linking environmental exposures to long-term alterations in gene expression (13). Epigenetic regulation acts as a dynamic interface between environment and gene expression, allowing external stimuli such as viral infections to induce long-lasting molecular changes in host cells. Among these mechanisms, DNA methylation, which consists of the addition of methyl groups to cytosine residues in CpG sites, is one of the most extensively studied epigenetic modifications and plays a key role in regulating immune and inflammatory responses. Changes in DNA methylation patterns have been reported following viral infections and may persist long after viral clearance, potentially contributing to long-term disease susceptibility (14, 15). A recent epigenome-wide study in infants with RSV infection has identified differential DNA methylation patterns associated with disease severity, supporting the role of epigenetic regulation in modulating host response to viral infection (16).

To date, genome-wide and epigenome-wide association studies (GWAS and EWAS) have identified >100 asthma-associated genetic variants and epigenetic markers, highlighting loci involved in immune regulation, inflammatory pathways, and epithelial function (17–25). Many early associations were identified in blood samples and have implicated pathways of Th2/Th1 immune signaling, eosinophilic inflammation, and antigen presentation. However, there has been a growing emphasis on tissue-specific biomarkers, particularly in nasal and airway epithelial cells, which lie at the interface between environmental exposures and airway immune responses (26, 27). In addition to DNA methylation, other epigenetic mechanisms such as histone modifications and non-coding RNAs (e.g., microRNAs) are increasingly recognized as modulators of gene expression in asthma, linking environmental exposures, immune and structural responses in the airway (28).

Given the substantial burden of both RSV infection and childhood asthma, the identification of biomarkers and early indicators of risk for long-term respiratory sequelae remains a key research priority. In this context, epigenetic profiling may provide valuable insights into the biological processes linking early viral infections with later disease (asthma) development.

## Epigenetic and gene expression links between early-life RSV infection and asthma in childhood

In our previous work (29), we investigated the epigenomic landscape of children hospitalized with RSV infection who were followed longitudinally for 5 years after the acute phase of the infection to evaluate the possible development of recurrent wheezing and/or asthma. Using genome-wide DNA methylation profiling, we identified multiple differentially methylated positions (DMPs) associated with the later development of respiratory sequelae. These epigenetic alterations were enriched in genes involved in inflammatory pathways and airway remodeling, suggesting that RSV infection may leave a persistent epigenetic signature influencing immune regulation and respiratory disease susceptibility.

Building on these observations, we explored whether DNA methylation biomarkers previously associated with asthma are also detectable in children who experienced RSV infection during infancy. To address this question, we examined genes previously implicated in epigenetic studies of pediatric asthma and evaluated their methylation and gene expression profiles in whole blood samples from our RSV cohort. These biomarkers were derived from the studies summarized in Table 1, which typically report DMPs or differentially methylated regions (DMRs) associated with asthma. In most cases, these studies identified significant CpG sites or genomic regions and provided annotation to nearby or associated genes. For the purpose of the present analysis, we compiled the genes mapped to CpG sites or regions linked to asthma and used them as candidate asthma-associated biomarkers.

**Table 1:** Overview of epigenetic studies and methylation platforms for the detection of asthma-associated biomarkers in children (*n* = 9).

Publication (Ref.)	Sample Type	Participants (sample size)	Platform
Yang et al. (17)	PBMCs	AC (97) vs. HC (97)	I450K
Chen et al. (18)	PBMCs	AC (97) vs. HC (97)	I450K
Jiang et al. (19)	WB & WBCs	Children and youth (5,147)	I450K
Reese et al. (20)	WB	Newborn (3,572) and children (2,862)	I450K
Arathimos et al. (21)	PB	Mother-child pairs (1,000)	I450K
Zhu et al. (22)	PBMCs & Tregs	ACh (15), HCh (15)	PyroS
Stefanowicz et al. (23)	PBMCs & AECs	HC (7), AT (9), AA (4), nAA (5)	IGG
Li et al. (24)	CB, CBMCs, BS	AC vs. Non-Case children. Meta-analysis (1,213)	EPIC
Pedersen et al. (25)	WB	Newborns (7,433)	EPIC

Sample types: PBMCs, peripheral blood mononuclear cells; WB, whole blood; WBCs, white blood cells; PB, peripheral blood; Tregs, regulatory T cells; ACEs, airway epithelial cells; CB, cord blood; CBMCs, cord blood mononuclear cells; BS, blood spot. Participants: AC, asthma cases; HC, healthy controls; ACh, asthmatic cases; HCh, healthy children; AT, atopic; AA, atopic asthmatic; nAA, non-atopic asthmatic. Methylation platform: I450: Illumina Infinium HumanMethylation450K BeadChip; PyroS, pyrosequencing; IGG, Illumina GoldenGate Methylation Cancer Panel I; EPIC, Illumina Infinium HumanMethylationEPIC BeadChip.

**Figure 1:**
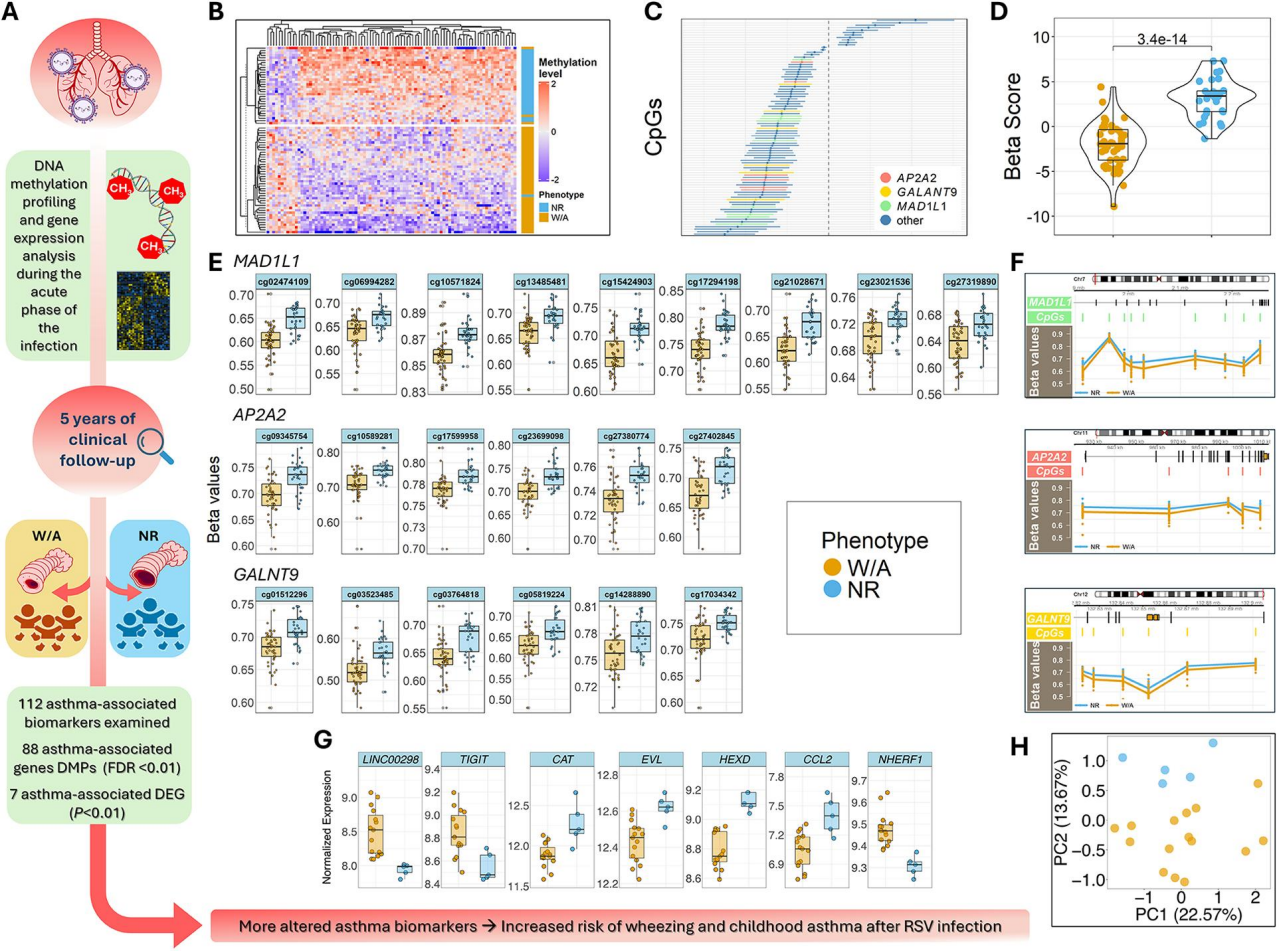
Integrated DNA methylation and gene expression signatures associated with wheezing/asthma outcomes. **(A)** study design: DNA methylation profiling and gene expression analyses were performed during the acute phase of infection, followed by 5-years of clinical follow-up to classify individuals into wheezing/asthma (W/A) and normal recovery (NR) groups. A total of 112 asthma-associated biomarkers were evaluated. **(B)** Heatmap of methylation levels for the candidate genes across all individuals. The color scale indicates methylation level (blue: hypomethylation; red: hypermethylation). **(C)** Forest plot showing the distribution of methylation effect sizes across analyzed CpG sites, illustrating the shift toward hypomethylation or hypermethylation. CpGs associated with the most frequent genes *MAD1L1*, *AP2A2*, and *GALNT9* are highlighted in green, pink, and yellow, respectively. **(D)** Violin plot showing the distribution of beta scores calculated using *GSVA* between W/A and NR groups. **(E)** Top: DNA methylation levels of the 9 CpG positions within the *MAD1L1* gene across sample groups. Middle: DNA methylation levels of the 6 CpG positions within the *AP2A2* gene across sample groups. Bottom: DNA methylation levels of the 6 CpG positions within the *GALNT9* gene across sample groups. **(F)** Genomic regional view of CpG methylation patterns in the *MAD1L1*, *AP2A2*, and *GALNT9* genes. The positions of CpG sites are shown along the genomic coordinates, together with the average methylation levels for NR and W/A groups. **(G)** Boxplots showing expression levels of the seven significantly differentially expressed genes (DEGs; adjusted *P*-value < 0.05) between W/A and NR groups. **(H)** Principal component analysis (PCA) of the expression of 92 out of the 112 asthma-associated biomarkers, showing a clear separation between W/A and NR individuals.

DNA methylation and gene expression levels were compared between two groups: children who later developed wheezing or asthma after RSV infection (W/A, *n* = 43; a subset of *n* = 15 with whole transcriptome data available) and those who recovered completely without respiratory sequelae (NR, *n* = 33; a subset [*n* = 5] with whole transcriptome data available) (Figure 1A). Whole-transcriptome data for the subset of 20 overlapping patients were generated and obtained from a previous study by Gómez-Carballa et al. (30). Gene expression data were normalized and corrected for sex and age differences. Following cell deconvolution analysis to infer the proportions of the main immune cell populations (data not shown), statistically significant differences between NR and W/A groups were observed only for plasma cells. Accordingly, gene expression data were further adjusted to account for differences in plasma cell proportions. A total of 9 studies, including meta-analysis or original data, were retrieved from the literature and reviewed (Table 1).

A total of 112 asthma-associated biomarkers were identified, with most showing hypomethylation patterns in children with asthma compared to healthy controls (Supplementary Table 1). For the methylation analysis, each gene was analyzed individually. Differences in DNA methylation between groups across CpG sites within each gene were assessed using a linear model adjusted for sex and age. Resulting *P-*values were corrected for multiple testing using the false discovery rate (FDR) method. CpG sites with an adjusted *P*-value < 0.01 were considered statistically significant.

This analysis identified 88 (*P*-value < 0.01; Supplementary Table 2) differentially methylated CpG sites across 49 of the 112 asthma-associated genes. When visualized across individuals using a heatmap (Figure 1B), the methylation profiles of these significant CpGs revealed a consistent tendency toward lower methylation levels in the W/A group. Similarly, the distribution of Delta Beta values (the difference in methylation levels) between W/A and NR confirmed that most of the observed changes corresponded to hypomethylation (Figure 1C). A z-score calculated from Beta values with Gene Set Variation Analysis (GSVA), considering all the significant positions together, supports this finding, showing a strong discrimination power between W/A and NR groups for the positions (Figure 1D). Notably, this pattern closely resembles findings from epigenetic studies of asthma in independent populations. Among the asthma-related genes analyzed, a large proportion have previously been described as hypomethylated in patients with asthma compared with healthy individuals (31). The concordance between these findings suggests that the epigenetic alterations observed in our RSV cohort may reflect biological pathways already implicated in asthma pathogenesis.

To examine these observations in greater detail, we focused on three genes showing particularly notable methylation patterns, namely, *MAD1L1* and *AP2A2*, and *GALNT9* all of which harbor multiple significant CpG sites. Figure 1E presents boxplots of the beta-values for individual CpG sites mapped to *MAD1L1*, *AP2A2*, and *GALNT9* loci, respectively, and Figure 1F provides a locus-level view of these methylation changes. For the three genes, 9 and 6 CpG sites distributed across each locus exhibit consistent differences in methylation between W/A and NR individuals. These patterns support the presence of locus-specific epigenetic alterations and suggest coordinated epigenetic regulation at the *AP2A2*, *MAD1L1*, *GALNT9* loci that may be associated with RSV-related outcomes.

Increased expression of *AP2A2* has been associated with asthma severity, exacerbations, and type-2 inflammatory biomarkers (21, 32). Genetic polymorphisms in the same gene have previously been associated with bronchitis and chronic obstructive pulmonary disease (COPD) (33). *AP2A2* encodes a component of the AP-2 endocytic complex involved in clathrin-mediated trafficking and has recently been involved in host–virus interactions during viral infection (34). It is therefore plausible that alterations in *AP2A2*-related pathways could influence both viral responses and airway inflammation. Similarly, *MAD1L1* has been implicated in studies investigating genetic and epigenetic determinants of respiratory and inflammatory diseases, such as asthma (35). Emerging evidence suggests that it may participate in pathways related to cell-cycle regulation and immune responses, potentially contributing to idiopathic pulmonary fibrosis (IPF) susceptibility (36). The last represented gene, *GALNT9* has recently been identified as differentially methylated in respiratory and inflammatory conditions. This gene encodes a glycosyltransferase involved in mucin-type O-glycosylation, a process relevant to epithelial barrier function and immune responses. These findings suggest that epigenetic alterations in *GALNT9* may also contribute to airway inflammation and asthma-related outcomes (27, 37).

Examination of gene expression profiles in the subset of our RSV cohort with available whole transcriptome data identified 92 of the 112 candidate genes in the dataset. A significantly different expression (adjusted *P*-value < 0.05; Supplementary Table 2) between W/A and NR groups was detected for seven of the candidate genes, namely *LINC00298*, *TIGIT*, *CAT*, *CCL2*, *EVL*, *HEXD*, and *NHERF1* (Figure 1G), with four genes downregulated and three upregulated in the W/A group. Notably, several of these genes showed highly significant differences (*P*-values as low as 0.00013), supporting robust transcriptional divergence between groups.

Principal component analysis (PCA) based on gene expression profiles of the 92 genes revealed a clear separation between W/A and NR samples, indicating distinct global expression patterns associated with asthma-related signatures (Figure 1H). These findings suggest that children who later develop wheezing or asthma after RSV infection exhibit coordinated alterations in gene expression, reinforcing the link between early transcriptional changes and increased risk of adverse respiratory outcomes.

Larger-scale studies will be needed to better define how the observed methylation differences functionally influence transcriptional regulation in the W/A and NR groups.

## Discussion

Despite growing interest in the long-term consequences of early-life viral infections, the relationship between RSV infection and epigenetic remodeling of the host methylome remains poorly characterized. Unfortunately, research in this area remains extremely limited, with only a handful of studies directly investigating the impact of early-life RSV infection on DNA methylation, other epigenetic modifications, or gene expression. Furthermore, current studies often suffer from small sample sizes, cross-sectional designs, and a lack of functional validation.

Preliminary observations suggest that some of the epigenetic pathways implicated in pediatric asthma may overlap with those activated during early RSV infection, including pathways involved in innate immune signaling, epithelial barrier function, and cytokine regulation, raising the possibility that viral exposure during critical windows of immune development could leave persistent molecular imprints. A central challenge for future research will be to determine whether RSV infection during early childhood can induce stable epigenetic modifications that contribute to long-term immune programming and increased susceptibility to asthma later in life. At present, however, it remains unclear whether the observed epigenetic signatures arise as a consequence of viral infection or instead reflect pre-existing susceptibility factors that predispose certain children both to severe RSV infection and to asthma development. Disentangling causality from correlation is therefore a key unresolved issue in the field. Addressing this question will require large, well-characterized prospective cohorts with sufficient statistical power and detailed clinical phenotyping, including standardized definitions of infection severity, environmental exposures, and asthma-related outcomes.

Equally important is the adoption of longitudinal, well-powered study designs that combine detailed immunophenotyping, repeated sampling, and the integration of multi-omic datasets, enabling the tracking of epigenetic trajectories from infancy through adolescence into adulthood, while capturing the dynamic interplay between viral exposures and host epigenetic programming. Studies incorporating biological samples collected before viral infection occurs would be particularly informative, as they could help distinguish whether specific methylation patterns precede infection or emerge as part of the host response to the virus. Birth cohorts with repeated sampling across early life may therefore provide a unique opportunity to clarify the temporal dynamics of epigenetic changes associated with viral exposures.

Furthermore, integrating methylome data with transcriptomic, immunological, and environmental information will be essential to unravel the specific causal relationships and to understand how early-life viral exposures interact with host genetic background and environmental factors. Advanced analytical frameworks, including multi-omics integration and systems biology approaches, will likely be required to interpret these complex datasets.

In this context, expanding analyses beyond peripheral blood to include multiple tissue types will be particularly important to capture tissue-specific epigenetic and transcriptional responses to infection. Non-invasive samples, such as buccal and nasal epithelial cells, represent especially promising alternatives, as they are readily accessible in pediatric populations and may more directly reflect airway-specific biological processes. Notably, we have recently identified DNA methylation signatures associated with severe RSV infection in infants using non-invasive buccal samples, supporting the feasibility and biological relevance of these approaches (16).

Such integrative approaches may reveal whether RSV-induced epigenetic marks represent transient responses to infection or durable molecular signatures of immune programming.

Clarifying this distinction will be critical for identifying biomarkers of disease risk and for developing preventive strategies aimed at reducing the long-term respiratory consequences of early-life viral infections. A deeper mechanistic understanding could inform targeted interventions, including vaccination strategies or early-life therapeutics, designed to modify disease trajectories before the onset of chronic respiratory conditions.

A limitation of the present study is that all included cases correspond to hospitalized RSV infections, which inherently excludes the large proportion of mild or asymptomatic infections managed in the community. Although our cohort includes a spectrum of disease severity, ranging from short hospital admissions to intensive care unit cases, the absence of non-hospitalized infections may limit the generalizability of our findings to the full spectrum of RSV disease. In addition, the relatively modest sample size, particularly for transcriptomic analyses, may limit statistical power to detect smaller effect sizes. While we adjusted for covariates such as age and sex, residual confounding factors cannot be fully excluded.

Taken together, the present perspective highlights both the promise and the current limitations of epigenetic research in RSV, emphasizing the need for robust, longitudinal, and mechanistically informed studies to clarify the role of early-life viral infections in shaping long-term respiratory health and to inform future preventive and therapeutic strategies.

## Data Availability

The original contributions presented in the study are included in the article/Supplementary Material, further inquiries can be directed to the corresponding author.
